# Animals Which Never See Daylight

**Published:** 1892-03

**Authors:** 


### ANIMALS WHICH NEVER SEE DAYLIGHT.

There are many animals in the world which pass all their lives in darkness, never seeing a ray of light. Every one has heard of the blind fishes of the Mammoth Cave. The latter is the biggest of 500 great caverns in the United States. All of them are inhabited by numerous other sorts of creatures that have no eyes for vision. Literally speaking, there is no such thing as a blind fish, since the most sightless of the finny tribe possesses visual organs in a rudimentary condition. But, through want of use, the optic ganglia and nerves have broken down and been absorbed.

Among the animals in these caves where Egyptian darkness ever dwells, are blind crayfish, colorless, which in the water by torchlight look like white phantoms of their outdoor kind. Now and then in such places one comes across a common frog, emaciated and seemingly discouraged, which has found its way, how no one knows, to the Tartarean realms. Also one discovers curious cave rats, of the same color as the domestic rats, but with longer bodies, like a weasel's, more developed whiskers and much bigger ears. Of bats there are multitudes in the caverns, as one might expect, inasmuch as they are creatures of darkness.

Spiders of several kinds are found in the caves. They are uniformly small, weak, and of sedentary habits. No webs do they spin, save a few irregular threads sometimes. What they live upon is rather a puzzle, though it is supposed that they catch stray mites and other such small fry. Scavengers constitute a large part of the population of the caverns. Carnivorous beetles are plentiful, particularly in those places where parties take lunch. The processes of decay seem to be accomplished briefly by a few fungi. It is said that meat hung up at the mouth of one of these caverns remains fresh for a long time, and it is surmised that the bacteria which causes things to become putrid are probably rare in the underground atmosphere.

No animals whatever are found in the dry parts of the caves. Dampness, or a certain degree of moisture, seems to be essential to their existence. Under the stones one finds white, eyeless worms, and in the damp soil around about are to be discovered blind beetles in little holes which they excavate and bugs of the thousand-leg sort. These thousand-leg bugs, which in the upper world devour fragments of dead leaves and other vegetable debris, sustain life in the caverns by feeding upon decayed wood, fungus growths and bats' dung. Kneeling in a beaten path one can see hundreds of them gathered about hardened drips of tallow from tourists' candles. There are plenty of crickets also.

So far as the insects of the cave are concerned, the loss of sight which they gradually undergo is sufficiently well understood. The first step is a decrease in the number of the facets which make up the compound eyes, with a corresponding diminishment of the lenses and retinæ. After four or five generations the eyes become useless. It would be most interesting to breed these or other blind creatures of the caves in the light, so as to find out if they would get their sight back. In all animals, including man, it is found that nature tries to compensate for loss of vision by increasing the power of the sense of touch. Thus the antenæ of cave insects grow remarkably long. It is very curious to find that nothing in their behavior suggests the fact that they are blind. They walk, run, stop, explore the ground and try to escape from the grasp of the bug hunter just if they they really saw. The light of a candle startles them as much as if they perceived it visually. It is a remarkable fact, proving that the ancestors of these creatures could see, that in the embyro stage of their existence they have eyes well developed.

In the abysses of the oceans, below 500 fathoms, many animals have either imperfect eyes or none. Their condition in this regard affords a suggestive parallel to that of cave life and the causes are probably the same. Science is of the opinion that all deep-sea life originally emigrated from the shallows. The creatures which dwelt in the darkness of the depths naturally lost their powers of vision after a while. It is the same way with the mole, which is doubtless descended from progenitors which could see. Blindness in the mole is the result of a degeneration of the optic nerve, the consequence of which is that images formed in the eye itself are not transmitted to the animal's consciousness. Occasionally a mole can see a little out of one eye which has retained its communication with the brain. It is not that the mole is born blind, but that it inherits a tendency to atrophy of the visual organs just as people derive from their parents an inclination to consumption or other diseases. Some day in the future there may be no such thing as a mole that is not entirely and hopelessly blind.

